# Supplementary material for: Quantification of early nonpharmaceutical interventions aimed at slowing transmission of Coronavirus Disease 2019 in the Navajo Nation and surrounding states (Arizona, Colorado, New Mexico, and Utah)
Source: PLOS Glob Public Health. 2023 Jun 21;3(6):e0001490. doi: 10.1371/journal.pgph.0001490 (PMC10284412; doi:10.1371/journal.pgph.0001490)
Supplement: Attachment — Submitted filename: Plos One Global Healthesponse_to_Reviewers.docx [file pgph.0001490.s001.docx]

**Response to Reviewers:**

Revision of manuscript PGPH-D-22-02023

**Quantification of early nonpharmaceutical interventions aimed at slowing transmission of Coronavirus Disease 2019 in the Navajo Nation and surrounding states (Arizona, Colorado, New Mexico, and Utah)**

Point by point responses are provided below. Changed/new text is highlighted in red.

Reviewer #1: In the summary part, the IMRaD structure must be respected. Break down each part. The main objective of the study should be clearly stated. If possible, the main results in terms of quantification of the measures, which really show the measured impact of the interventions, should also be included.
I would therefore like to see the whole summary taken up again, taking into account the comments.

***Response 1.1:*** The Abstract is unstructured, consistent with journal conventions. The material in the Abstract covers background, methodology/principal findings, and conclusions/significance. We modified the Abstract to include a clearer statement of the study objective.

Here, we investigated these differences in disease transmission dynamics with the objective of quantifying the contributions of non-pharmaceutical interventions (NPIs) (e.g., behaviors that limit disease transmission).

In the Introduction, we added a statement of the study objective starting at line 80.

The objective of this study is to quantify the effect of early non-pharmaceutical interventions (NPIs) on the transmission of COVID-19 in the Navajo Nation (NN) and surrounding states: Arizona (AZ), Colorado (CO), New Mexico (NM), and Utah (UT).

The introduction failed to address the general aspect of the subject. It would therefore be better to shed light on the situation of approaches that have approached it in the same way in other countries outside your study framework. As a result, your discussion has stopped at mere comments. There is really no discussion under the subheading discussion of results with those of other authors discussion.

It would be really interesting to compare your work with what other authors have done elsewhere. If not present, mention it too.

***Response 1.2:*** We revised the Discussion to include references to studies of NPIs in China and the UK (Refs. 34 and 35).

A generalizable result that we obtained is that sustained adherence to NPIs can prevent surges in disease incidence whereas relaxation of NPIs can lead to surges in disease. Our results add evidence in support of the idea that the duration of NPI adherence is an important factor influencing their effectiveness [34, 35].

Do not mix the methods and introduction sections

***Response 1.3:*** Please see Response 2.2.

Why, is it directly in the results section that the objective of the study was first stated? Is it a result? I don't think so. I would like to see it brought back to the introduction of the abstract part and the end of the introduction part of the body of the article.

***Response 1.4:*** The objective of the study was originally stated at the beginning of the Results section. We have moved this material to the Introduction as well as stating the objective more clearly in the Abstract (see Response 1.1).

Reviewer #2: Comments
Dear authors have submitted a manuscript entitled "Quantification of early nonpharmaceutical interventions aimed at slowing transmission of Coronavirus Disease 2019 in the Navajo Nation and surrounding states (Arizona, Colorado, New Mexico, and Utah)". This manuscript is the product of Bayesian inference-based modeling and reports a very interesting finding that the effectiveness of NPIs lies in the fact that they are sustained measures rather than measures per se. This is very interesting research, however, there are limitations, especially methodological ones, which should be corrected before the manuscript is accepted.

***Response 2.1:*** We are gratified that the reviewer found our study to be “very interesting.” Methodological limitations are addressed below.

Introduction
In general, the introductory section is well written and groups the most important aspects to provide context to the research.

However, in the last paragraph, between lines 93 and 103, the objective of the research is stated, and a brief "summary" of the methodology used is added. In the context of the form of the manuscript this is not correct. Please replace the text from line 93 to 103 with the proposed overall research objective of the manuscript.

***Response 2.2:*** Lines 93-103 in the original Introduction section have been moved to the Methods section. This material can now be found starting at line 246. The study objective statement at the start of the original Results section has been moved to the Introduction section. This material can now be found starting at line 80.

Methodology
Dear authors, the materials and methods section, while describing useful and important information for the manuscript, has important deficiencies that must be addressed before the manuscript can be accepted for publication.
Although this is a modeling-based study, it is recommended that the section be restructured according to STROBE guidelines as soon as the methodology allows, including the sections on:
Study design
Setting
Exposure
Outcome
Data sources
Model calculation and modeling process

For example, the text between line 105 and 125 should be added to the "Data sources" section.
The description of the modeling performed is quite complete and I consider that the analysis methods are correct, however, the description as with the rest of the text in the methodology section should be structured in the corresponding section "Model calculation and modeling process". Please, in cases where equations are used make sure that these are described after an end point using the corresponding function to provide greater understanding to the reader.
As mentioned above, please make sure to add or restructure the necessary information to cover the mentioned sections.

***Response 2.3:*** We have restructured the Methods section for consistency with STROBE guidelines. The Methods section now consists of the following subsections: 1) Study design, populations, and setting; 2) Data collection and sources; 3) Study bias; 4) Model and simulations; 5) Data analysis; and 6) Ethical considerations.

The material from lines 105 to 125 in the original manuscript are now located in the Data collection and sources subsection of Methods starting at line 136.

Results
Please move the research objective to the end of the Introduction section.
Likewise, move the text from lines 208 to 213 to the corresponding section in the Methods section.
For the rest, the findings proposed in the results section should be objective to avoid confusion among readers, the figures are adequate and of quality I consider that they do not require changes.

***Response 2.4:*** We have moved material as requested. The research objective is now found in the Introduction section (please see response 2.2). The material from lines 208-213 in the original manuscript are now located in the Data analysis subsection of Methods starting at line 246. Subjective terms in the Results section have been eliminated.

Discussion
Line 282 to 289: Initially the limitations of the study should be mentioned in the final paragraph of the discussion section. On the other hand, they claim the limitation of not being able to determine the relative efficacy of NPIs equally, please add a logical argument for this limitation.
Also, while it is true that they mention that the model does not consider other variables that could make it more accurate, this is a correct assertion. However, regarding the possible association with the emergence of new SARS-CoV-2 variants, despite not being able to perform complex statistical analyses, you could conduct a descriptive analysis of variant circulation in the Navajo Nation and surrounding cities to weigh your findings against variants that were shown to have higher transmissibility rates than the native variant such as the Omicron variant. If you do not have access to this information, please add a detailed description of your limitation in the appropriate section.

***Response 2.5:*** We moved the material discussing the limitations of the study from lines 282-289 in the original manuscript to the penultimate paragraph of the discussion section beginning at line 402. In this paragraph we provide a clearer explanation of why we could not estimate the efficacy of individual NPIs.

Our approach for detecting changes in disease-avoiding behaviors has at least two limitations. First, we cannot ascertain the relative effectiveness of individual NPIs. The reason is that our model only accounts for the overall effect of all NPIs. In the model, persons are either protected by NPIs or not.

In the first paragraph of the Discussion we now explain that variants of concern did not emerge during the period that we focused on in our study (see lines 342-346).

In our model, we do not consider multiple strains of SARS-CoV-2 because during the time period of interest (21-January-2020 to 14-September-2020) variants of concern had yet to emerge. The first variant of concern (Alpha, B.1.1.7) appeared in the United Kingdom (UK) in late September of 2020 [32] and was detected in the US in November 2020, which is beyond the time window of our study [33].
